# Cost-effectiveness analysis of MRI, CE-CT and 18F-FDG PET/CT for detecting colorectal liver metastases eligible for hepatic resection

**DOI:** 10.3389/fonc.2023.1161738

**Published:** 2023-07-24

**Authors:** Moritz L. Schnitzer, Niklas von Münchhausen, Gloria Biechele, Jasmin Runtemund, Freba Grawe, Thomas Geyer, Clemens G. Kaiser, Florian Haag, Johannes Rübenthaler, Matthias F. Froelich

**Affiliations:** ^1^Department of Radiology, University Hospital Munich, Ludwig-Maximilians-University Munich, Munich, Germany; ^2^Department of Radiology and Nuclear Medicine, University Medical Centre Mannheim, Medical Faculty Mannheim-University of Heidelberg, Mannheim, Germany

**Keywords:** cost-effectiveness, CRLM, PET/CT, MRI, hepatic resection

## Abstract

**Objectives:**

Colorectal cancer (CRC) is a serious challenge for the health system. In 2022 CRC represented 8% of cancer diagnoses in the United States. 30% of patients already show metastases at the initial tumor staging. The majority of these metastases are sited in the liver. According to their extension and the status of the tumor colorectal liver metastases can be treated in several ways, with hepatic resection being the gold-standard. Contrast-enhanced computed tomography (CE-CT), positron emission tomography/computed tomography (PET/CT) and magnetic resonance imaging (MRI) can be used for evaluation of resectability of these liver metastases. The aim of this study is to assess the most economic imaging modality for detecting liver metastases eligible for hepatic resection by analyzing their cost-effectiveness.

**Materials and methods:**

In our study, a Markov state transition model was built to calculate the quality-adjusted life years (QALYs) and overall costs for each diagnostic strategy in accord with the stated input values obtained from scientific research. Further, probabilistic sensitivity analyses by means of Monte Carlo simulations were performed to consider possible model uncertainties. For evaluation of the cost-effectiveness on an economic threshold, the Willingness-to-pay (WTP) was set at $ 100,000. The applied values and the calculated results are based on the U.S. healthcare system.

**Results:**

CE-CT led to overall costs of $ 42,874.02 and 8.47 QALYs, whereas MRI led to $ 40,863.65 and 8.50 QALYs. PET/CT resulted in overall costs of $ 43,216.74 and 8.48 QALYs. Therefore, MRI was determined to be the dominant strategy in the model. According to the performed sensitivity analyses, MRI remained cost-effective over a wide range of WTPs.

**Conclusion:**

In conclusion, according to our analysis, MRI is the dominant strategy for detecting hepatic metastases eligible for hepatic resection in colorectal cancer.

## 1 Introduction

Colorectal cancer (CRC) poses a significant challenge to global health, as it is one of the most prevalent cancer types in the world. In the United States, CRC accounts for approximately 8% of newly diagnosed tumors and 9% of all cancer-related deaths (1). Risk factors for CRC include family history, metabolic diseases such as diabetes and obesity, chronic inflammatory intestinal diseases, and the use of nicotine and alcohol (2–4). Notably, the incidence of CRC in individuals under the age of 50 has increased significantly over the past few decades (5). Approximately 50% of CRC patients develop metastases during the course of their disease, with 26.5% of these metastases occurring in the liver (6, 7).

Fortunately, curative therapy for Colorectal liver metastases (CRLM) is achievable, and a complete remission can be achieved. Patients with untreated metastases have a median three-year overall survival of 27.5% (8). The gold standard for the treatment of liver metastases is surgical resection, which is recommended as the standard procedure for R0-resectable metastases in the new 2022 ESMO guidelines (9). However, due to a poor health status of the mainly elderly patients or inconvenient metastatic location near important liver structures, approximately 80% of patients are still not suitable for surgical resection (10). Therefore, it is essential to select liver metastases that are suitable for surgical resection during the diagnostic process. For liver lesions that are not eligible for resection, the most common treatment options are thermal ablation methods such as microwave ablation (MWA), radiofrequency ablation (RFA), or cryotherapy. The gold standard in CRLM treatment is currently under contention as the COLLISION Trial, which compares thermal ablation to surgical resection in the presence of a resectable and ablatable liver lesion (10).

An accurate and timely diagnosis is critical for identifying metastases accurately and selecting the most appropriate treatment for the patient, which can improve survival rate and overall health (11). In addition, proper imaging is vitally important for Follow-Up of the patients, as the local recurrence rates of liver metastases may reach to 55-60% (12).

Contrast-enhanced computed tomography (CE-CT), magnetic resonance imaging (MRI), and positron emission tomography/computed tomography (PET/CT) with 18F-fluorodeoxyglucose (18F-FDG) as a tracer are the preoperative imaging modalities for detecting CRLM (13). MRI is in fact superior to 18F-FDG PET/CT and CE-CT for detecting liver metastases due to its better soft-tissue contrast with contrast-agencies (14). Despite the diagnostic value of each imaging method, the monetary value of the investigated strategies still needs to be examined. The goal of this article is to estimate the long-term cost-effectiveness of MRI, CE-CT, and 18F-FDG PET/CT for detecting CRLM eligible for hepatic resection in relation to each other.

## 2 Materials and methods

### 2.1 Markov model design

**Figure 1:**
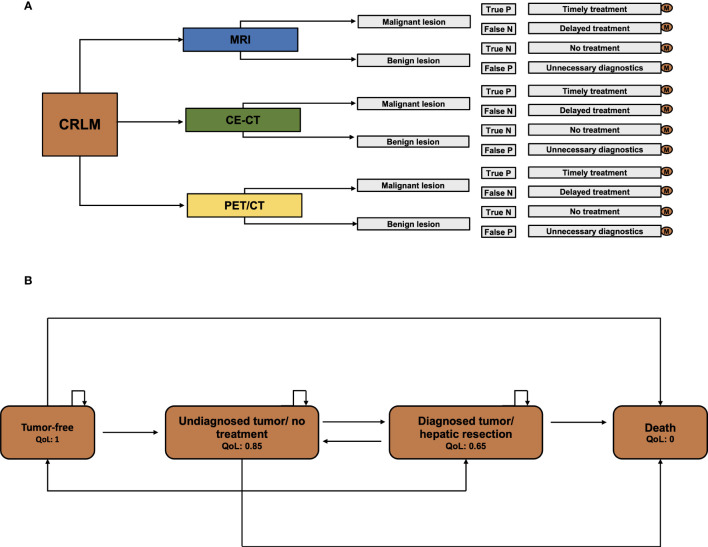
Model scheme. **(A)** Decision model for CE-CT, PET/CT, and MRI. For every single pathway, separate Markov calculations were executed. **(B)** The Markov model with the specified health stages “tumor-free”, “Undiagnosed tumor/no treatment”, “Diagnosed tumor/hepatic resection”, and “Death”.

To evaluate the financial value of imaging techniques for identifying CRLM suitable for ablation, a decision analysis was conducted using TreeAge Pro 2021 (Williamstown, MA) software. A Markov model was employed to forecast the long-term outcomes of patients based on the chosen imaging approach. A Markov model is a statistical tool that estimates the probabilities of all predefined model states and transitions between states in a complex system (**Figures 1A, B**). The model includes the model states “tumor-free”, “diagnosed tumor/no treatment”, “diagnosed tumor/hepatic resection”, and “death”. At the start of each measured cycle - in our model every cycle is one year for an overall duration of 5 years - the patient’s model state transits to a different state according to preset probabilities. At every moment in the model, the patient can be sorted into one of the preset model states. Each model state can also be associated with specific preset expenses and quality of life.

### 2.2 Input values

According to guidelines for executing cost-effectiveness analyses, costs and utilities are discounted by 3.00%. Additionally, Willingness-to-pay (WTP) is set to $ 100,000 per quality-adjusted life-year (QALY). The WTP can be seen as a limit of costs that the healthcare system of a society is willing to pay for a certain health profit. The mean age of the patient undergoing diagnostics for CRLM was 68 years in accordance with CRLM collectives. The applied values and the calculated results are based on the U.S. healthcare system. An overview of the applied input values is given in **Table 1**.

**Table 1:** Input values.

Name	Estimate	Distribution	Source
Expected value at diagnostic procedure	68	**β**	Engstrand et al., 2018 (6)
WTP	$ 100,000.00		Sanders et al., 2016 (15)
Discount for costs and utilities	3.00%		Sanders et al., 2016 (15)
Markov Model time frame	5 years		Sanders et al., 2016 (15)
Diagnostic test performances			
CE-CT sensitivity	65.70%	**β**	Sivesgaard et al., 2018 (14)
CE-CT specificity	93.65%	**β**	Sivesgaard et al., 2018 (14)
MRI sensitivity	84.85%	**β**	Sivesgaard et al., 2018 (14)
MRI specificity	92.05%	**β**	Sivesgaard et al., 2018 (14)
PET/CT sensitivity	72.05%	**β**	Sivesgaard et al., 2018 (14)
PET/CT specificity	92.85%	**β**	Sivesgaard et al., 2018 (14)
Costs (Acute)			
CE-CT	$ 464.00	�	Medicare (74177)
MRI (contrast-enhanced)	$ 964.00	�	Medicare (74183, 72197)
PET/CT	$ 1,615.00	�	Medicare
Cost of hospital stay (per day)	$ 2,606.00	�	Henry J Kaiser Foundation, KFF.org
Hepatic resection costs	$ 4.450.00	�	Medicare
Days in hospital	7		NG KKC et al., 2017 (16)
Overall resection costs	$ 21,592.00	�	Medicare
Delayed resection, further tests	$ 28,069.60	�	Expert opinion (1.3x as expensive)
Costs (Long Term)			
Annual expenses without tumor	$ 0	�	Assumption
Annual expenses with active CRLM	$ 63,063.00	�	Chen et al., 2018 (17)
Utilities			
QOL of patients without tumor	1	**β**	Assumption
QoL after resection	0.78	**β**	Wiering et al., 2011 (18)
QoL with recurrence	0.65	**β**	Kim et al., 2016 (19)
QoL with undetected recurrence	0.85	**β**	Assumption
Death	0		Assumption
Transition probabilities			
Risk of death without tumor	(age dependent)	**β**	US Life Tables 2015
Probability of successful treatment	70%	**β**	Expert opinion
Probability of recurrence after resection	62%	**β**	Hirokawa et al., 2019 (20)
Probability of hepatic metastases	27.50%	**β**	Engstrand et al., 2018 (6)
Probability of death without treatment	24.17%	**β**	Siebenhüner et al., 2020 (8)

#### 2.2.1 Diagnostic accuracy

The sensitivity and specificity values of the imaging methods in question are based on a European Radiology published article from Sivesgaard et al., 2018 comparing the diagnostic accuracy of CE-CT, MRI and 18F-FDG PET/CT (14). The sensitivities of CE-CT, 18F-FDG PET/CT, and MRI are therefore 65.70%, 72.05%, and 84.85%, whereas the specificities are 93.65%, 92.85%, and 92.05%. These values are averages of the diagnostic accuracy of the two reader results in the study.

#### 2.2.2 Utilities, costs and probabilities

Utilities are assessed as quality-adjusted life years (QALYs) as a value of the patients’ health status in every model state.

The costs of the imaging methods in question were obtained from Medicare in 2023. The costs for each modality may increase in future, as they undergo a yearly increase of around 10% (21). These increases of costs were not considered in the analysis. In addition, the costs for hepatic resection and the hospital stay after hepatic resection for every day were added to the analysis. Moreover, false negative imaging results that lead to a delayed treatment were estimated to be 1.3 times as high as a treatment in time.

The probability for a patient without tumor, for a diagnosed tumor without a treatment started, for a diagnosed hepatic tumor with surgical treatment and for death were incorporated in the model. To estimate the probability of the patient’s demise for any other reason than a tumor-related, US Life Tables were utilized as a reference. Additionally, the values for the probabilities for changing between the model states were assessed from scientific literature.

### 2.3 Economic analysis

QALYs and overall costs were calculated in the base-case scenario and customized in accordance with the applied discount rates and the Willingness-to-Pay. Further, incremental cost-effectiveness ratios (ICER) were calculated. The ICER is a parameter that measures the economic value of a diagnostic strategy and is calculated by the following formula:


ICER = (e1 −e0 )/(q1−q0)

In the ICER-formula, e1 and e0 are describing the cumulative short- and long-term costs of each diagnostic strategy, whereas q1 and q0 are describing the utilities and therefore effectiveness of each diagnostic strategy. The value of the ICER stands for the additional cost per QALY for each diagnostic strategy.

### 2.4 Sensitivity analysis

To simulate the influence of input parameter changes on imaging strategies’ cost-effectiveness, a deterministic sensitivity analysis was performed. The analysis altered overall costs and diagnostic accuracy within a reasonable range to highlight their impact. A tornado diagram was used to display the ICERs after various changes.

Meanwhile, a probabilistic sensitivity analysis was conducted to investigate the general uncertainty of input parameters and their effect on cost-effectiveness. Using probability distributions, a Monte Carlo data simulation was carried out with 50,000 iterations to assess the model results’ overall stability.

### 2.5 CHEERS statement

The fundamental basics of the methodology are based on the Consolidated Health Economic Evaluation Reporting Standards (CHEERS) statement. The major criteria of the checklist on how to perform cost-effectiveness analyses are met in this study (22, 23).

## 3 Results

### 3.1 Cost-effectiveness analysis

**Figure 2:**
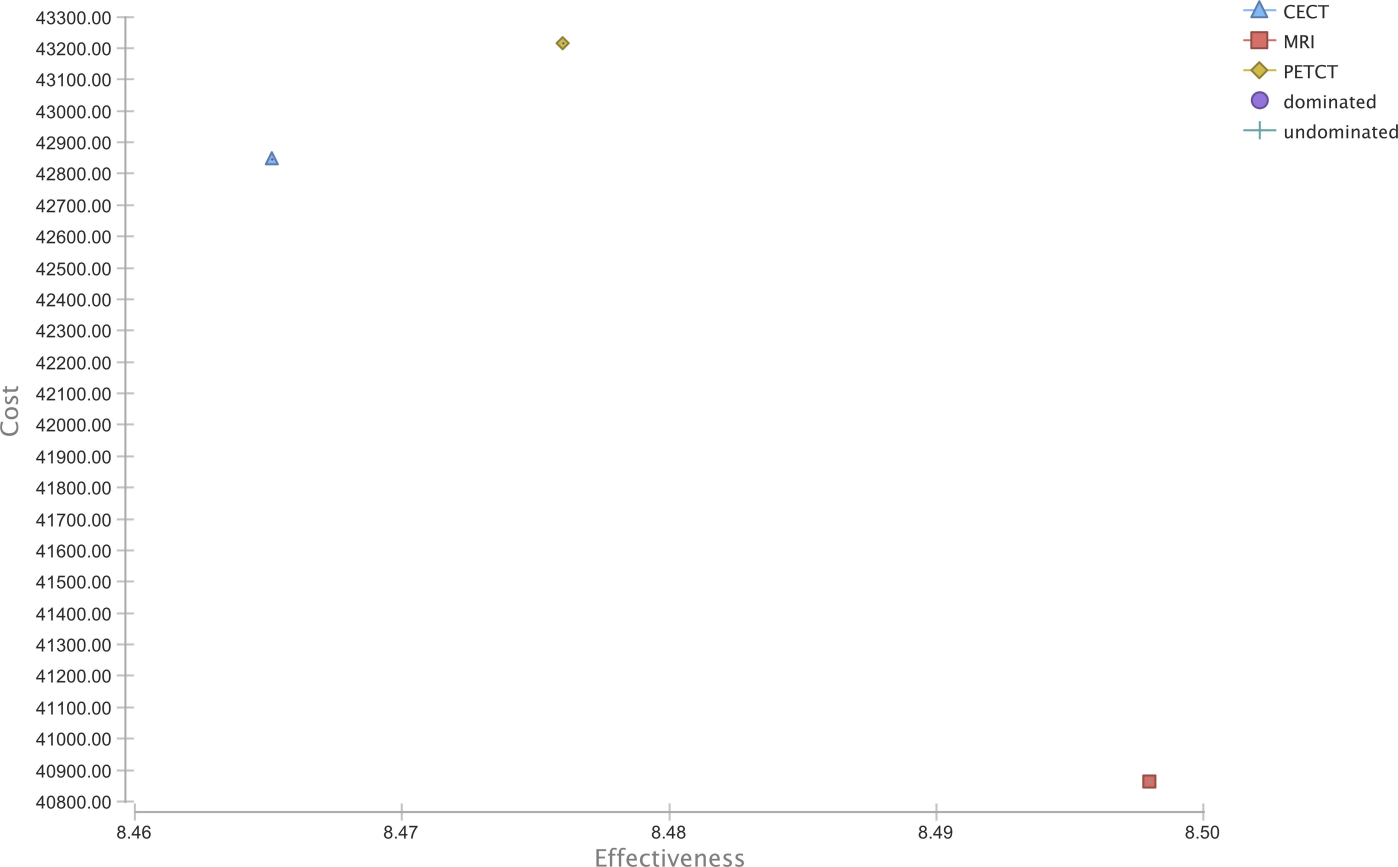
Cost-effectiveness analysis.

The strategies MRI, CE-CT, and PET/CT generated overall costs of $ 40,863.65, $ 42,874.02, and $ 43,216.74 with the effectiveness of 8.50, 8.47, and 8.48 QALYs in the baseline calculations with a WTP of $ 100,000. As a result, MRI dominated CE-CT and PET/CT in overall costs as well as in its effectiveness. The cost-effectiveness ranking is shown in **Figure 2**.

### 3.2 Sensitivity analysis

**Figure 3:**
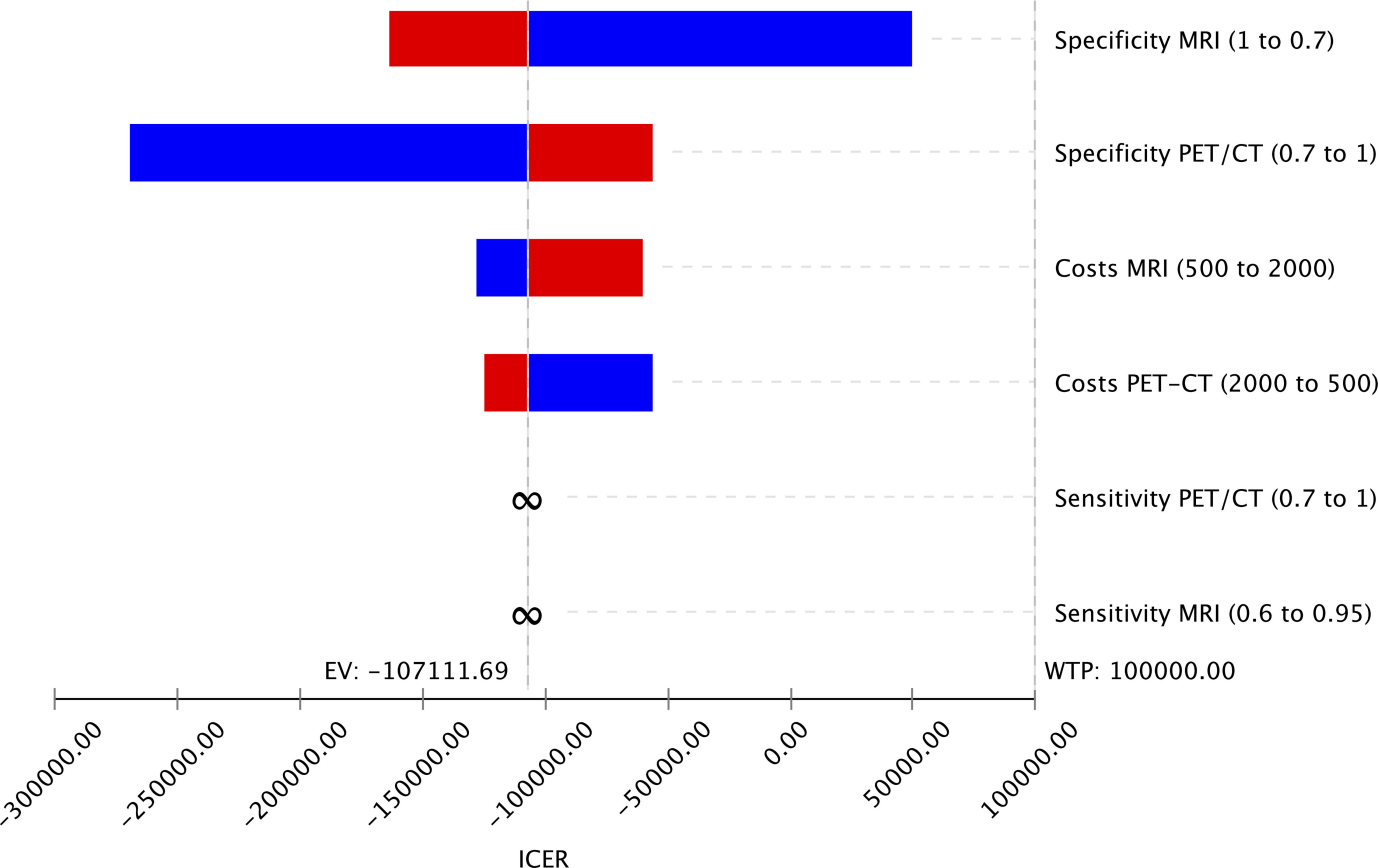
Tornado Diagram displaying variable changes of input parameters on the cost-effectiveness of the imaging strategies MRI and PET/CT showing that the specificities of both imaging methods have the highest impact on the cost-effectiveness.

The study investigated how changes in input parameters affected the cost-effectiveness of different imaging strategies using deterministic sensitivity analysis. The results are presented in a Tornado Diagram (**Figure 3**), which shows that the specificities of MRI and PET/CT have the most significant impact on cost-effectiveness. However, since MRI is cheaper and more effective than PET/CT, even changes in the input parameters within the range tested did not significantly affect the cost-effectiveness of MRI.

**Figure 4:**
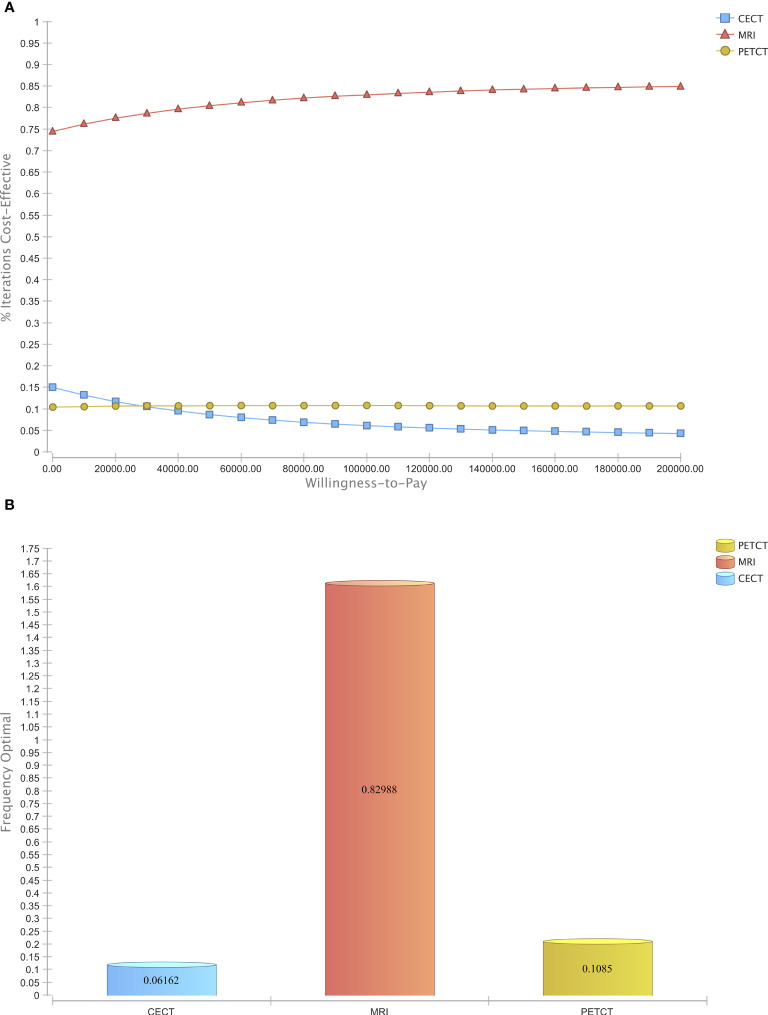
Probabilistic sensitivity analysis. **(A)** Acceptability Curve visualizing the economic dominance of MRI in the majority of reiterations over a wide span of WTPs **(B)** Acceptability at Willingness-to-Pay of $ 100,000.

To assess the general uncertainty of the input parameters and their influence on cost-effectiveness, the study used a Monte Carlo Simulation with 50,000 iterations. Across a broad range of costs, MRI was found to be the most cost-effective modality compared to CE-CT and PET/CT in the majority of iterations (**Figure 4A**). Furthermore, when considering a willingness-to-pay threshold of $100,000, MRI was the cost-effective modality in 82.99% of the simulations, whereas CE-CT and PET/CT were cost-effective in only 6.16% and 10.85%, respectively (**Figure 4B**). These results, based on 50,000 patient cases, demonstrate the economic superiority of MRI over CE-CT and PET/CT and suggest that MRI may be the preferred strategy for detecting CRLM eligible for hepatic resection.

## 4 Discussion

Our model reveals the cost-effectiveness of MRI for detecting CRLM eligible for hepatic resection compared to CE-CT and 18F-FDG PET/CT. MRI offers - alongside its economic superiority - the advantage of having the most reliable diagnostic accuracy compared to CE-CT and PET/CT. In addition to its economic advantages, MRI offers superior diagnostic accuracy compared to CE-CT and PET/CT, with the Diffusion-Weighted Imaging and T2-weighted fat suppression sequences being crucial for ensuring the highest accuracy. MRI also has an advantage for detecting recurrence of local metastases (24, 25). For instance, Sakai et al. (2022) discovered that the fat signal fraction in MRI after hepatic resection is associated with local recurrence. Therefore, due to the technological features of MRI, local recurrence can be detected earlier than in CE-CT (26). Furthermore, the importance of MRI especially for the clinical management of CRLM was proved in a meta-analysis of Vreugdenburg et al., 2016. This meta-analysis including 13 studies with 1025 patients on the one hand shows the diagnostic superiority of MRI over CE-CT with sensitivity values ranging from 86.9 to 100% for MRI and from 51.8 to 84.6% for CE-CT, and on the other hand it demonstrates that MRI had a significant influence on the clinical management of CRLM in 16.8% of patients with prior CE-CT. The fact that 1 of 6 patients is able to get a better treatment only through an additional MRI is huge and endorses the importance of MRI for treatment planning (27). In addition, it must be emphasized that most important driver for cost-effectiveness is not the cost of imaging, but more the costs for the treatment and the potential retreatment of a heavier tumor burden caused by an insufficient diagnostic workup in the first place, meaning that even if the costs for certain imaging methods vary in the real world, it does not have a strong influence on the cost-effectiveness outcome as the costs for imaging are just a small fraction compared to the overall costs of surgery and ongoing treatment. In order to minimize the risk of such a scenario, a diagnostic workup with MRI as the best imaging method for this indication is recommended that the patient can have the best possible treatment available and even has the chance for being cured.

In the baseline scenario, the cost-effectiveness of MRI is quite stable. In order to determine where a possible breaking point for CE-CT may be, we ran a supplementary 2-way deterministic sensitivity analysis (**Supplementary Figure 1**). On the vertical axis the sensitivity of CE-CT is shown, whereas the horizontal axis represents the sensitivity of MRI over a wide range. The colored areas represent the cost-effectiveness of each modality. As one can see, there may be a breaking point to CE-CT if sensitivity values of CE-CT increases unproportionally whit simultaneously decreasing sensitivities for MRI. With a stable sensitivity value for MRI over 85%, a breaking point for CE-CT is only a hypothetical one, as an increase of CE-CT sensitivity beyond 85% may be unrealistic. Nonetheless, the technological improvement in CT technology with Photon-counting CTs may change the outcome in the future. Therefore, it may become interesting to reevaluate the results when the diagnostic performance of photon-counting CT has been investigated over a larger patient number.

Another issue that needs to be acknowledged are disappearing liver metastases (DLM) due to preoperative chemotherapy. In 7 to 48% of cases of patients with neoadjuvant chemotherapy before resection of the liver metastases, these metastases become undetectable by CE-CT and CE-MRI after their chemotherapy cycles. However, an invisibility in imaging does not necessarily correlate with pathological remission. This can lead to overseen metastases during the primary resection and result in rising recurrence rates after resection, as not every single metastasis is targeted in therapy. Surgical studies recommend that even if the metastases disappeared in imaging, they should still be resected. Disappearing liver metastases on the one hand are still macroscopically visible during surgery at 25-45%, but on the other hand, the successfully treated metastases do have a recurrence rate of 50-80%. This may be another field where PET-MRI may have a significant impact on future clinical practices, which is to be discussed later in this article (28, 29).

Although MRI was cost-effective in 82.99% of repeats in the baseline calculations and was very stable even after alterations in the sensitivity analyses, it is vitally important to recognize some limitations. Like any model, the results are heavily reliant on the input parameters used. While we sourced most of our data from reputable scientific sources, these sources may not accurately reflect daily clinical reality, which could affect the results. Additionally, we consulted experienced physicians for their expert opinion on some input parameters without any previously published data to ensure accuracy. Further, the results of our model are based on the U.S. healthcare system. The results may deviate depending on the healthcare system of each country and cannot be blindly applied to all countries in the world. Nonetheless, with minor adjustments tailored to each respective healthcare system, this model is adaptable to most western industrialized nations.

It is worth noting that Saing et al. (2018) published an article on cost-effective imaging methods for resectable liver metastases, in which they compared the economic value of contrast-enhanced MRI (CE-MRI) and CE-CT and found CE-MRI to be cost-effective. However, their study did not consider PET/CT as a diagnostic modality, which offers significantly better diagnostic accuracy than CE-CT. Therefore, the cost-effectiveness of diagnostic modalities for CRLM for resectable liver metastases needs to be reconsidered. Nonetheless, our investigation showed that MRI is still the most cost-effective modality even compared to 18F-FDG PET/CT, strengthening MRI’s position as the most economic modality for detecting CRLM eligible for hepatic resection (30).

Despite MRI’s cost-effectiveness, there may be situations where a physician encounters patients with MRI examination contraindications, such as some cardiac pacemakers or metallic foreign bodies. Under these circumstances, the most economic examination method would have to be disregarded. In such cases, alternative imaging methods should be considered. On the one hand, PET/CT offers superior outcomes than CE-CT, but it comes with a significantly higher radiation dose. Therefore, we recommend individual decisions for every patient as the improved diagnostic and long-term treatment outcomes may outweigh any potential long-term effects of higher radiation exposure for a severe illness such as CRLM (31).

Over the last years, PET/MRI as an upcoming diagnostic modality has caused quite a stir in imaging of many tumor entities. Despite its higher costs and its limited availability, PET/MRI offers many advantages over PET/CT. It combines the supreme soft tissue contrast of MRI and the versatility of functional imaging in PET and offers a significant reduction of radiation dose. Nonetheless, the future role of PET/MRI in broad clinical reality is still unsettled (32, 33). Yet, studies proved the value of PET/MRI in imaging of CRLM, as PET/MRI offers a significantly higher diagnostic accuracy with 96.1% compared to 18F-FDG PET/CT with 82.4% for detecting liver metastases (34–36). According to Zhou et al., 2021, a one-stop protocol with 18F-FDG PET/CT combined with an abdominal PET/MRI has an significant impact on the choice of therapeutic management of liver metastases (37). Further, a one-stop 18F-FDG PET/MRI protocol is reported to be a valid diagnostic workup for rectal cancer staging (38). In another study, FDG-PET/CT was compared to pelvic MRI and abdominal and thoracic CT for detecting synchronous distant metastases in rectal cancer. The investigation proved PET/MRI to be clearly superior compared to a MRI and CT workup not only for lymph nodes and hepatic lesions, but as well for pulmonary lesions, which is a weak point of MRI for staging of CRC (39). Overall, PET/MRI offers a broad range of possibilities and advantages. Yet, the lack of availability and the costs speak against a widespread use of PET/MRI. Nevertheless, Gassert et al., 2021 proved the cost-effectiveness of 18F-FDG PET/MRI with hepatocyte-specific contrast agent for M-staging of rectal cancer compared to conventional staging workup (40). The most relevant and unstable factor for cost-effectiveness in this study was in fact the costs for PET/MRI. This indicates that if PET/MRI gets used more often in clinical practice and the costs for every singular procedure decrease, it may become a serious competitor to the currently established imaging modalities. However, to really prove the rentability of PET/MRI on a larger scale, there needs to be deeper investigation in further studies.

## 5 Conclusion

In conclusion, MRI can be considered the cost-effective strategy for detecting liver metastases eligible for hepatic resection and should therefore be seen as the modality of choice in the diagnostic workup routine.

## Data availability statement

The original contributions presented in the study are included in the article/**Supplementary Material**. Further inquiries can be directed to the corresponding author.

## Author contributions

MS, JRü, MF, and CK contributed to conception and design of the study. MS, NM, and MF performed the economic modelling. MF, MS, GB, FG, JRun, TG, FH, and JRü contributed to input data collection. MS, NM, and GB wrote the first draft of the manuscript. MF, MS, GB, FG, JRun, TG, FH, CK, and JRü edited the first draft and made final adjustments. All authors contributed to the article and approved the submitted version.
